# Antibacterial electrospun nanofibrous mats fabricated from polycaprolactone, gelatin and *Synedrella nodiflora* extract for biomedical applications

**DOI:** 10.1039/d6ra01137c

**Published:** 2026-05-19

**Authors:** Amanullah, Mainul Islam, Md. Mustafizur Rahman, Md. Farhad Hossain

**Affiliations:** a Dhaka University of Engineering and Technology Gazipur-1707 Dhaka Bangladesh 22205008@student.duet.ac.bd; b School of Textile Science and Engineering, Wuhan Textile University Wuhan China; c Department of Textile Engineering, Daffodil International University Dhaka-1216 Bangladesh mainulislam.nandail@gmail.com mustafizbutex95@gmail.com farhaddiu2807@gmail.com

## Abstract

Skin injuries are highly prone to bacterial infection and slow healing that necessitate the use of advanced wound dressings with effective antibacterial effect, appropriate morphology and appropriate moisture management. We aimed to fabricate and evaluate the antibacterial electrospun nanofibrous mats of varying concentrations of polycaprolactone (PCL), gelatin (GEL) and *Synedrella nodiflora* (SN) extract for biomedical applications. Scanning electron microscopy (SEM) showed that uniform, bead-free nanofibers were obtained with a fiber diameter decreasing from about 247 ± 12 nm to about 207 ± 12 nm, in the control and extract-loaded mat, respectively. Fourier transform infrared (FTIR) spectroscopy confirmed incorporation of the SN extract into PCL/GEL nanofibers through the appearance of extract-specific functional groups and characteristic peak shifts, indicating strong intermolecular interactions without covalent crosslinking. Thermogravimetric analysis revealed a single-stage thermal degradation behavior of the developed PCL/GEL/extract nanofibrous mat, indicating enhanced thermal stability and improved structural integrity compared with those of the individual polymer components. The nanofibrous mats exhibited adequate tensile strength and elongation at break, indicating good mechanical stability and flexibility. The moisture management test revealed enhanced absorption, one-way transfer of moisture and improved ability to handle moisture in extract-loaded mats. *In vitro*, an initial moderate release was followed by sustained release, indicating controlled delivery without a significant burst effect. The Kirby–Bauer disk diffusion antibacterial test revealed that the control sample had no inhibitory effect, while the extract-impregnated mats exhibited a substantial antibacterial role against Gram-positive and Gram-negative bacteria, with an inhibitory zone diameter of 25.7 ± 0.6 and 30.3 ± 0.6 mm, respectively. Cytotoxicity analyses further verified an excellent biocompatibility and cell viability of >95%. Collectively, the developed PCL/GEL/SN electrospun nanofibrous mats could be used as antibacterial and biocompatible wound dressing materials.

## Abstract 2

Electrospun PCL/gelatin nanofibers incorporating *Synedrella nodiflora* extract showed enhanced antibacterial activity, controlled release, and improved moisture management, making them promising candidates for wound healing applications.

## Graphical abstract

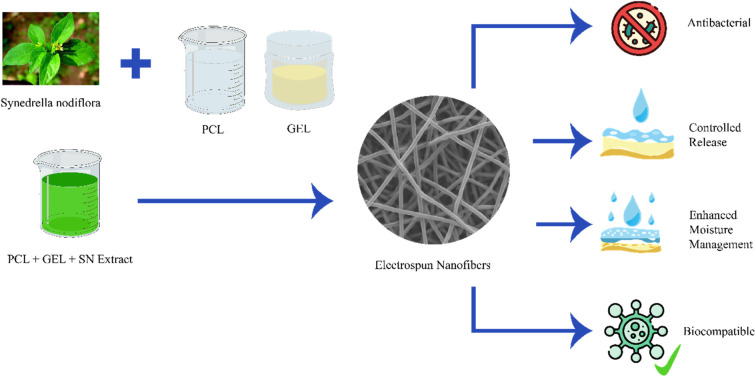

## 1 Introduction

The skin is among the largest and one of the most essential organs of the human body. It serves as a protective barrier against environmental variables, viruses, and physical harm.^1–3^ It is critical for maintaining homeostasis by regulating temperature and preventing fluid loss.^4,5^ Skin injuries caused by trauma or burns are highly susceptible to microbial infection; therefore, wound dressings with effective antibacterial activity are required to protect the wound site and support the healing process.^6,7^ As a result, improved wound healing is very important to restore the integrity and functionality of the skin. Wound healing is a cascade process involving hemostasis, inflammation, proliferation, and maturation. First, the body reacts by clotting after an injury to halt bleeding.^8,9^ The second stage is the inflammatory stage, in which immune cells cause the removal of dead tissue and combat infections. In the proliferation stage, new tissue is formed, which consists of collagen, and the remodeling of this tissue is encouraged in the final stage to enhance the strength of the wound site.^10,11^ However, several complications, such as infection, delayed healing, and scarring, may undermine the healing process. Effective wound care products are required that could contribute to and speed up the healing process.^12–14^

A wound dressing is an important part of the healing process because it helps to protect the wound against external contaminants, ensuring a moist environment, and enabling tissue regeneration.^15–17^ However, conventional dressings may provide limited antibacterial activity, drug-release control, and extracellular matrix-like support. Therefore, nanofiber-based dressings are promising due to their high surface area, porous structure, and extracellular matrix-like morphology.^17,18^ Nanofiber-based wound dressings have become promising options because of their high surface area and porous structure, and they resemble the natural extracellular matrix. All these properties enable nanofiber dressings to support cell growth, provide controlled release of drugs, and provide enhanced antibacterial protection.^19–22^

The use of bioactive agents^23–27^ or synthetic antimicrobial agents in nanofiber wound dressings can enhance the functional characteristics of the dressing to a great extent.^28,29^ These agents can treat infections, stimulate the regeneration of tissues, and enhance healing.^30,31^ The antimicrobial properties of nanofibers have been demonstrated to be increased by incorporating silver nanoparticles or plant-based antimicrobial compounds into nanofibers, resulting in decreased chances of wound infection and a better healing process. These developments have made nanofiber-based wound dressings effective solutions for medical applications.^32–35^

*Synedrella nodiflora* (SN) has antibacterial and antioxidant properties, which have been attributed to the presence of bioactive compounds, including phenolic acids and flavonoids.^36^ SN is a plant with notable medicinal properties and could be integrated into wound care products.^37–40^ Research has demonstrated that the extracts of the SN leaves have strong effects on a diverse variety of pathogens.^36,41,42^ This plant-based antimicrobial effect can be used in the creation of nanofiber-based dressings to decrease the risk of infection and enhance the healing process.

Polycaprolactone (PCL) is a biodegradable and biocompatible polymer. It plays an important role in tissue engineering and drug delivery systems.^43,44^ Due to its suitable mechanical characteristics and electrospinning convenience, it is commonly used to produce nanofibers. Nonetheless, this hydrophobic property of PCL can restrict its moisture management in wound dressings, which may interfere with the healing process.^45,46^ Alternatively, gelatin (GEL) is a natural polysaccharide that has numerous applications in cell culture and wound healing because it can form hydrogels, which enhance cell proliferation and tissue regeneration.^47^ It is an excellent complement to PCL due to its hydrophilic properties. GEL, when combined with other materials, increases the hydrophilicity of the composite material, which is useful in trapping moisture and the overall healing of wounds. Also, GEL is a hemostatic agent that further promotes the regeneration process, making it an ideal choice for wound dressing.^48,49^ The biocompatibility, biodegradability, and improved moisture management of PCL- and GEL-based nanofibers make a significant contribution to the improvement of healing.^50,51^ Besides, PCL-GEL nanofiber-based dressings show enhanced flexibility, enhanced water retention, better cell migration and tissue growth. These improved characteristics render PCL-GEL nanofibers the most suitable option for advanced wound care.^52^

Despite the promising potential of PCL- and GEL-based wound dressings, there has been limited research on a combination of PCL, GEL, and plant extracts, particularly extracts of SN leaves. Studies have shown that the moisture-management performance of electrospun wound dressings is typically evaluated by the water contact angle, swelling/water uptake, and water vapor transmission behavior.^53^ Within this context, PCL provides structural stability but is inherently hydrophobic, whereas GEL improves hydrophilicity and exudate affinity.^54,55^ Therefore, PCL/gelatin hybrid nanofibers are widely regarded as promising wound dressing matrices because they provide a better balance between mechanical integrity and moisture handling than PCL alone.^56^ Similar trends have been reported in plant-functionalized PCL/GEL systems, including clove oil, cinnamon, *Pinus radiata* extract, *Gymnema sylvestre* extract, and arbutin-loaded nanofibers,^57–61^ where the added phytochemicals further contributed to wettability, antibacterial performance, and wound-healing functionality.^24^

SN is of particular interest because its extracts have been reported to possess antibacterial, antioxidant, and anti-inflammatory activities,^62–64^ which are all relevant to infected-wound management. Widely used plant extracts such as aloe vera, curcumin, and green tea have been extensively investigated for their established antioxidant and antimicrobial properties.^65–67^ However, SN offers a less explored but multifunctional phytochemical source.^68^ On that basis, SN was selected in the present study as a bioactive additive for PCL/GEL nanofibers to investigate whether it could provide antibacterial functionality while retaining the favorable scaffold characteristics of the polymeric matrix.

**Fig. 1:**
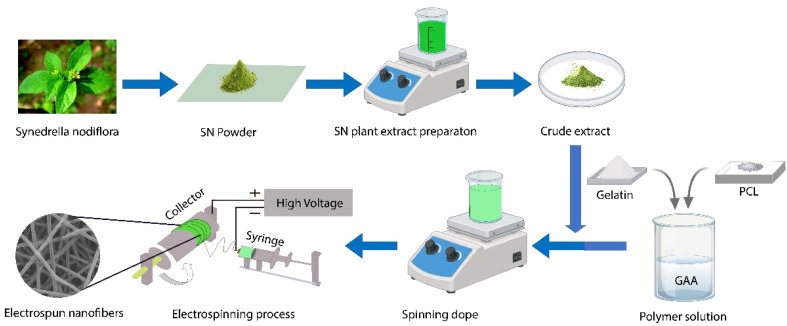
Preparation of SN extract and fabrication of extract-loaded PCL/GEL electrospun nanofibrous mats (schematic).

In the present study, electrospun nanofibrous mats were developed using PCL and GEL polymers, incorporating SN plant extract to impart bioactive functionality. The morphology and structural characteristics of fabricated nanofibers were examined using scanning electron microscopy (SEM) to evaluate fiber uniformity and surface features. Fourier transform infrared (FTIR) spectroscopy was employed to confirm the presence of the selected polymers, the incorporation of the plant extract, and to identify potential chemical interactions within the nanofibrous matrix. The moisture-handling performance of the developed mats was assessed through moisture management testing (MMT) to determine their absorbency and liquid transport behavior. Thermogravimetric analysis (TGA) was conducted to evaluate the thermal stability and degradation profile of samples. The mechanical properties of the nanofibrous mats were evaluated using tensile testing to determine their tensile strength and elongation at break. *In vitro* release studies were performed to analyze the release behavior of SN extract from the nanofibrous mats using UV-vis spectrophotometry. The antimicrobial efficacy of the nanofibrous mats was investigated using the Kirby–Bauer disk diffusion method, with antibacterial performance quantified by measuring the zones of inhibition against selected pathogenic microorganisms. Finally, cytotoxicity assays were performed to assess the biocompatibility and potential toxicity of the developed nanofibrous mats, thereby determining their suitability for biomedical applications. The fabrication process is summarized in Fig. 1.

## 2 Materials and methods

Fresh leaves of SN were collected from Netrakona (Dhaka, Bangladesh). PCL with a molecular weight of 70 000 to 90 000 (catalog number: 440744) was purchased from Sigma–Aldrich (St. Louis, MO, USA). GEL (catalog number: G1890) was procured from Sigma–Aldrich (Germany). Glacial acetic acid (GAA; catalog number: 100063), an organic solvent sourced from VWR (Darmstadt, Germany), was used to dissolve PCL and GEL. Ethanol (catalog number: 8074) was utilized for the plant extract and was sourced from Junsei Chemicals (Beijing, China).

### 2.1 Preparation of plant extract

**Fig. 2:**
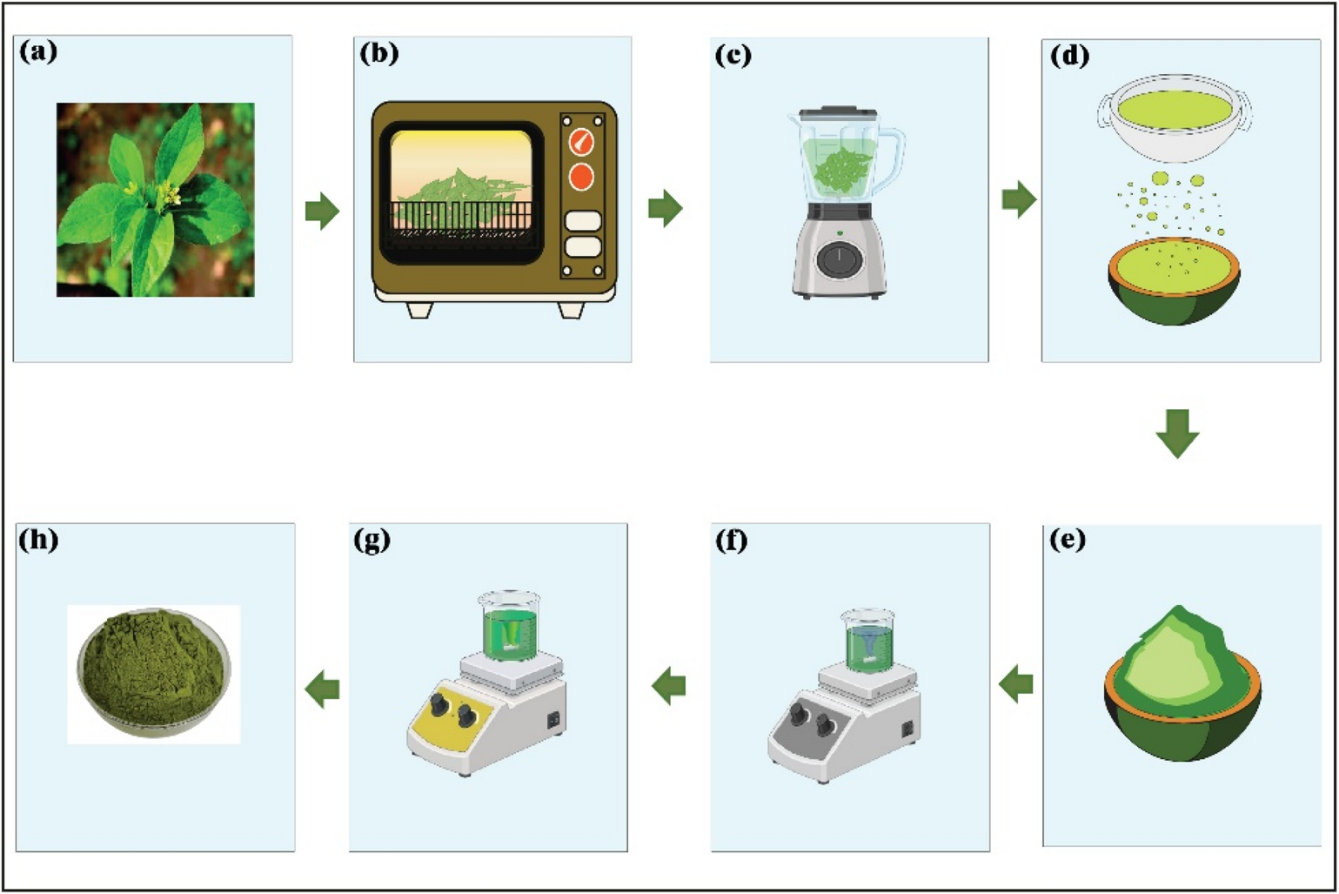
Extraction process of SN (schematic). (a) Fresh SN leaves, (b) oven drying, (c) grinding, (d) sieving, (e) SN leaf powder, (f) extraction process, (g) solvent evaporation, and (h) crude extract.

Leaves were washed with water to remove dirt and then dried in the shade for 5 days. The dried leaves were then ground into a fine powder using a mechanical grinder and kept in an airtight container. To prepare the extract, 400 g of SN leaf powder was mixed with ethanol at a solvent-to-solid ratio of 1 : 3 (*w/v*) and agitated using a magnetic stirrer for 24 h to ensure efficient extraction of bioactive compounds. The resultant extracts were filtered twice using a multilayered nylon mesh, subjected to a magnetic stirrer, and evaporated at 80 °C until all the solvent had been removed. The extracts were stored at 4 °C to be used in mat preparation. Fig. 2 is a schematic of the plant extraction process.

### 2.2 Preparation of mats

To produce PCL-GEL electrospun nanofiber mats, GEL powder (5%, *w/v*) was initially dissolved in GAA (90%, *v/v*) at 45 ± 2 °C for 4 h. After the complete dissolution of GEL, PCL pellets (12%, *w/v*) were added to the solution and stirred overnight at 25 ± 2 °C. The polymer solution in GAA remained at a final concentration of 16% (*w/v*). Three separate 30 mL solutions were prepared for electrospinning. The first solution, labelled “Pure PG”, contained only PCL and GEL, with no extract added. PGSN-1 was the second sample that contained 8 mg of SN extract powder, which was added to the PCL-GEL solution. Conversely, the third sample, which was named “PGSN-2”, had 12 mg of the SN extract powder blended in the PCL-GEL solution.^69^ A magnetic stirrer was used to combine the solutions thoroughly by stirring them at 60 ± 2 °C after the addition of the extract to achieve a homogeneous mixture.^70^ They were then used in the electrospinning of the nanofiber mats. Table 1 displays the ratios of the three samples and sample compositions.

**Table 1:** Composition of PCL/GEL and SN extract in different samples

Sample	PCL + GEL (ml)	SN extract (mg)
Pure PG	30	0
PGSN-1	30	8
PGSN-2	30	12

### 2.3 Electrospinning

Electrospinning was done in a custom-built single-needle electrospinning apparatus. The system was made up of a negative high-voltage power supply that made contact with a 10 mL syringe filled with the spinning solution. The earth connection was used as the ground (0 kV). A high positive voltage was applied to the needle during electrospinning.

A jet was started with a 20-gauge metallic needle. A 30 kV voltage was applied to generate a stable Taylor cone, and this started the generation of nanofibers. A syringe pump was used to pump a flow rate of 1.2 mL h^−1^ of the solution. The distance between the tip of the needle and the grounded collector was adjusted to 14 cm. We used a rotating drum collector (diameter: 158 mm; length: 500 mm) operating at 500 rpm to maintain uniform fiber deposition and to minimize bead formation during electrospinning. Electrospinning was performed for 8–10 h to produce homogenous nanofibrous mats, which were then used for further evaluation and use.

### 2.4 Characterization

#### 2.4.1 Scanning electron microscope (SEM)

The morphological and structural characterization of the electrospun nanofibrous mats was carried out using Scanning Electron Microscopy (SEM) (Model SU 1510; Hitachi, Japan) to determine the fiber diameter, porosity, and uniformity of nanofibers, which are essential variables that determine the performance of the materials in biomedical applications.^71^ Prior to imaging, samples were sputter-coated with a thin layer of gold (Au) to enhance conductivity and minimize charging effects during SEM. Fiber diameters were measured from SEM micrographs using ImageJ software by analyzing randomly selected fibers from multiple images to ensure statistical reliability.

#### 2.4.2 Moisture measurement test (MMT)

To measure the dynamic liquid transport characteristics by the samples in terms of wetting, absorption, spreading, and one-way moisture transport, the Moisture Management Test (MMT) (Model: M290; SDL Atlas, UK) was conducted. Major variables such as wetting time, rate of absorption, speed of spreading, Accumulative One-Way Transport Index (AOTI), and overall moisture management capacity (OMMC) were tested. This test provides quantitative analysis of the capacity of materials to control liquid moisture under simulated conditions of skin contact. MMT results were also interpreted according to AATCC standard guidelines.^72^

#### 2.4.3 Antibacterial assay

To determine the antibacterial activity of three electrospun nanofiber mats (Pure PG, PGSN-1, and PGSN-2), the Kirby–Bauer method was used. Mueller–Hinton agar plates were inoculated with a bacterial suspension that was prepared by dispersing bacteria in sterile saline solution. The bacterial suspension was adjusted to a concentration of 1.0 × 10^5^ CFU mL^−1^ using the 0.5 McFarland standard to ensure uniform bacterial density for the antibacterial assay. A sterilized cotton swab was used to evenly apply the bacterial suspension on the agar plates. Discs of 6 mm in diameter were taken out of each of the nanofiber mat samples and placed on the surface of the inoculation plates with sufficient separation between them. The plates were incubated at 37 °C for 24 h, after which the inhibitory zone around each disc was measured in millimeters. Inhibition of the size of the zones was measured to determine the antibacterial efficiency of each sample.^73^

#### 2.4.4 Fourier transform infrared (FTIR)

Fourier Transform Infrared spectroscopy (FTIR) (Model: IRPrestige21, Shimadzu Corporation, Japan) was used to examine the chemical composition and functional groups of the developed electrospun nanofibrous mats. FTIR spectroscopy was used to confirm the existence of esters, alcohols, and carboxyl functional groups, which are important in comprehending the reaction between the polymer skeleton and SN extract. FTIR spectroscopy was done in the range 4000–400 cm^−1^ to identify the typical peaks that belonged to different functional groups in the nanofibers.^74^

#### 2.4.5 Thermogravimetric analysis (TGA)

The thermal stability and degradation properties of the developed electrospun nanofiber mats were analyzed by thermogravimetric analysis (TGA) on the SDT 650 system (Discovery, USA). TGA measurements were performed under a nitrogen atmosphere with a flow rate of 50 mL min^−1^. Samples were heated from 30 °C to 600 °C at a constant heating rate of 10 °C min^−1^. TGA was used to investigate the effects of adding SN extract on the thermal properties of the mats. The weight loss of samples was recorded over the selected temperature range to evaluate their thermal degradation behavior. Results provide information regarding the thermal stability, degradation temperature, and possible interactions between the polymer matrix and incorporated extract.^75^

#### 2.4.6 Cytotoxicity assay

The cytotoxicity test was performed using Vero cell lines at Waffen Research Laboratory (Dhaka, Bangladesh) in a biological safety cabinet (NU400E; Nuaire, USA), with incubation carried out in a CO_2_ incubator (Nuaire, USA). Cell morphology and viability were evaluated using a trinocular microscope equipped with a camera (Optika, Italy) in conjunction with a hemocytometer. The culture of African green monkey kidney epithelial cells was done in Dulbecco's Modified Eagle Medium (DMEM) supplemented with 1% streptomycin-penicillin (1 : 1), 0.2% gentamicin, and 10% fetal bovine serum (FBS). Cells were inoculated at a density of 1.5 × 10^4^ cells per 100 µL in 48-well plates and incubated for 24 h at 37 °C in a 5% CO_2_ atmosphere. Wells were then inoculated using sterilized (autoclaved) samples. Cell viability was determined using a hemocytometer-based cell counting method after appropriate staining to distinguish viable and non-viable cells. Cytotoxic effects after 48 h of exposure were analyzed using an inverted microscope, and cell viability was quantified by hemocytometer-based counting. Cell viability (%) was calculated as (number of viable cells/total cells) × 100. Experiments were done in duplicate to ensure reproducibility.^76^

#### 2.4.7 Tensile properties

The tensile properties of the electrospun nanofibrous membranes were measured using a universal testing machine (Instron 3345) at a crosshead speed of 10 mm min^−1^ under ambient conditions. Rectangular samples (50 × 10 mm^2^) were tested under uniaxial loading until failure. Each sample was tested three times, and average values are reported.

#### 2.4.8 Extract release behavior

Two extract-loaded nanofibrous samples (PGSN-1 and PGSN-2) were immersed in 11 mL of phosphate-buffered solution (PBS, pH 7.4) and incubated at 37 °C. At predetermined time intervals, 2 mL of the release medium was withdrawn and replaced with an equal volume (2 mL) of fresh PBS to maintain a constant volume. Collected samples were analyzed using UV-vis spectrophotometry in a wavelength range of 260–350 nm, and the maximum absorbance wavelength (*λ*_max_) of the SN extract was used to evaluate release behavior.

#### 2.4.9 Statistical analyses

Quantitative data were analyzed and are presented as the mean ± standard deviation to ensure accurate representation of measurements. The results obtained from different tests were expressed as average values of repeated measurements. Additionally, single-factor ANOVA was applied for statistical evaluation.

## 3 Results and discussion

### 3.1 Morphological analyses

The electrospun nanofiber mats made of PCL-GEL and SN extract exhibited different fiber diameters. Inclusion of the plant extract played a part in determining the fiber diameter. The pure PG scaffold appeared white/off-white and maintained a relatively uniform fibrous structure. In contrast, the PG-SN scaffolds exhibited a brownish coloration due to the incorporation of SN extract, which contains naturally occurring phytochemical compounds such as polyphenols and flavonoids responsible for the observed color change.

**Fig. 3:**
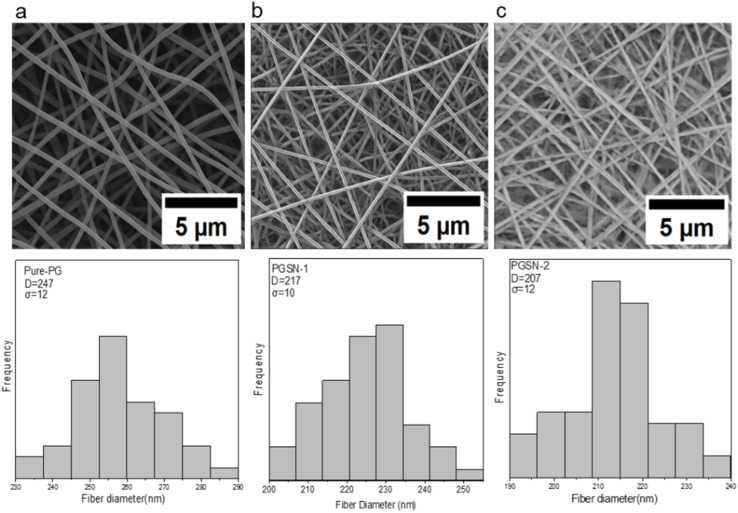
Scanning electron micrographs and corresponding fiber diameter distributions of developed nanofibrous samples: (a) pure-PG, (b) PGSN-1, and (c) PGSN-2.

The Pure PG sample, containing only PCL and GEL, exhibited an average fiber diameter of 247 ± 12 nm, as observed from SEM. Fiber diameters were determined by measuring 50 randomly selected fibers from the scanning electron micrographs using ImageJ, and the results are expressed as mean ± standard deviation (Fig. 3). This diameter can be explained by the typical parameters of electrospinning and the polymer composition, in which the PCL/GEL ratio can influence the solution behavior and the resulting fiber formation during electrospinning.

Comparatively, PGSN-1 and PGSN-2 samples showed a slightly reduced mean fiber diameter of 217 ± 10 nm and 207 ± 12 nm, respectively. One-way ANOVA revealed a highly significant difference among the groups (*p* < 0.01), indicating that the incorporation of SN extract influenced the fiber diameter significantly. The decrease in fiber diameter when SN extract was added to the solution suggested that the plant extract may have altered the electrospinning solution properties, particularly conductivity, which can influence fiber formation. The other compounds present in the extract may have altered the behavior of the solution during electrospinning to allow the fabrication of thinner fibers. This observation aligns with research that has indicated identical effects in cases where bioactive compounds are incorporated into polymer solutions.^77^

The declining nature of the fiber diameter as the concentration of the extract (PGSN-1 to PGSN-2) increased was also indicative of the varying concentration of the extract on the electrospinning process. A higher extract concentration is associated with the formation of finer fibers, which may be related to changes in the electrospinning solution behavior. Moreover, the presence of bioactive compounds in the extract of SN could have a positive influence on the electrospinning process, lowering the surface tension or increasing the electrostatic interaction between the solution and collector.

### 3.2 FTIR spectroscopy

**Fig. 4:**
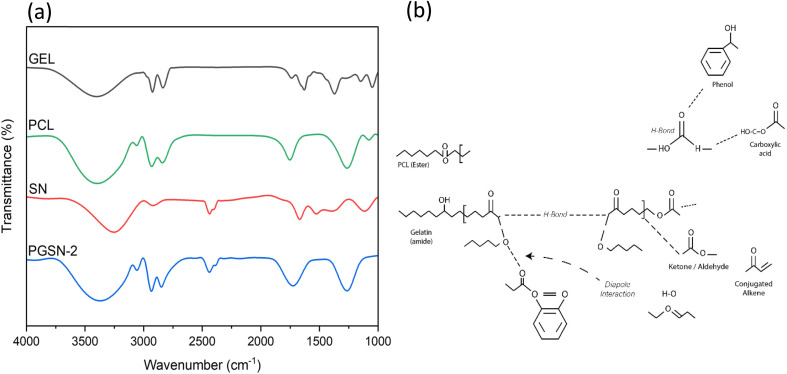
(a) FTIR spectra of GEL, PCL, SN extract, and PGSN-2 nanofibers; (b) schematic representation of intermolecular interactions among PCL, GEL, and SN extract.

The inclusion of PGSN-1 and PGSN-2 resulted in significant changes in the FTIR spectra, indicating incorporation of the extract, as shown in Fig. 4a. In the pure PCL and GEL, characteristic peaks for PCL included 1724 cm^−1^ for C=O stretching of the ester bond, 2927 cm^−1^ and 2859 cm^−1^ for C–H asymmetric and symmetric stretching (sp^3^), and 1251 cm^−1^ for C–O and C–C stretching.^78^ GEL exhibited characteristic peaks at 3251 cm^−1^ for N–H stretching of the amide, 1653 cm^−1^ for C=O stretching of amide I, and 1521 cm^−1^ for N–H bending of amide II.^79^ Upon adding SN extract, several new peaks appeared, including 1384.56 cm^−1^ for phenol, 1381.81 cm^−1^ for aldehyde, 1462.94 cm^−1^ for alkaline methylene groups, and 1737.48 cm^−1^ for the lactone group. Additionally, peaks at 1164.96 cm^−1^ for sulphonamide, and the broad band around ∼3400 cm^−1^ was assigned to overlapping O–H and N–H stretching vibrations, indicating the presence of hydroxyl and/or amine groups. The peak at 1630.36 cm^−1^ was attributed to the conjugated alkene. The absorption peak observed at 1710 cm^−1^ was attributed to the C=O stretching vibration of carbonyl groups, which may have included contributions from ester or carboxylic functionalities. The C=O stretching peak of PCL at 1724 cm^−1^ broadened and shifted to the range of 1724 cm^−1^ to 1737 cm^−1^ due to interactions between the polymer and extract. The emergence of such discrete peaks was testimony to the incorporation of the functional groups of the PGSN-1 and PGSN-2 nanofiber mesh. Therefore, this was a testament to the incorporation of PGSN-1 and PGSN-2.

A proposed schematic depicting the molecular interactions within PCL/GEL nanofibers containing the extract, derived from the corresponding FTIR spectral variations, is shown in Fig. 4b. The system represents a physically blended nanofibrous matrix without the use of covalent crosslinking agents. PCL contributes ester carbonyl (C=O) groups, characterized by an intense absorption band at ∼1724 cm^−1^, while GEL provides amide and N–H functional groups typical of protein-based polymers. Intermolecular hydrogen bonding between the carbonyl groups of PCLs and the N–H groups of GELs is likely to occur, contributing to changes in the intensity and broadening of the N–H (3200–3500 cm^−1^) and carbonyl (1700–1750 cm^−1^) regions of the FTIR spectra.

The SN extract is represented by key phytochemical functional groups, including phenolic hydroxyl, carboxylic acid, lactone, ketone/aldehyde carbonyl, and conjugated alkene moieties. These groups interact with the PCL/GEL nanofiber matrix predominantly through secondary intermolecular interactions such as hydrogen bonding and dipole–dipole attractions rather than covalent bonding. Hydrogen bonding between the hydroxyl or carboxylic acid groups of the extract and the carbonyl groups of PCLs or N–H groups of GEL, along with dipole interactions among polar carbonyl functionalities, accounts for the emergence of new absorption bands and the broadening or slight shifting of the PCL carbonyl peak. These spectral changes confirmed the incorporation of the SN extract within the nanofibrous matrix.

### 3.3 TGA

**Fig. 5:**
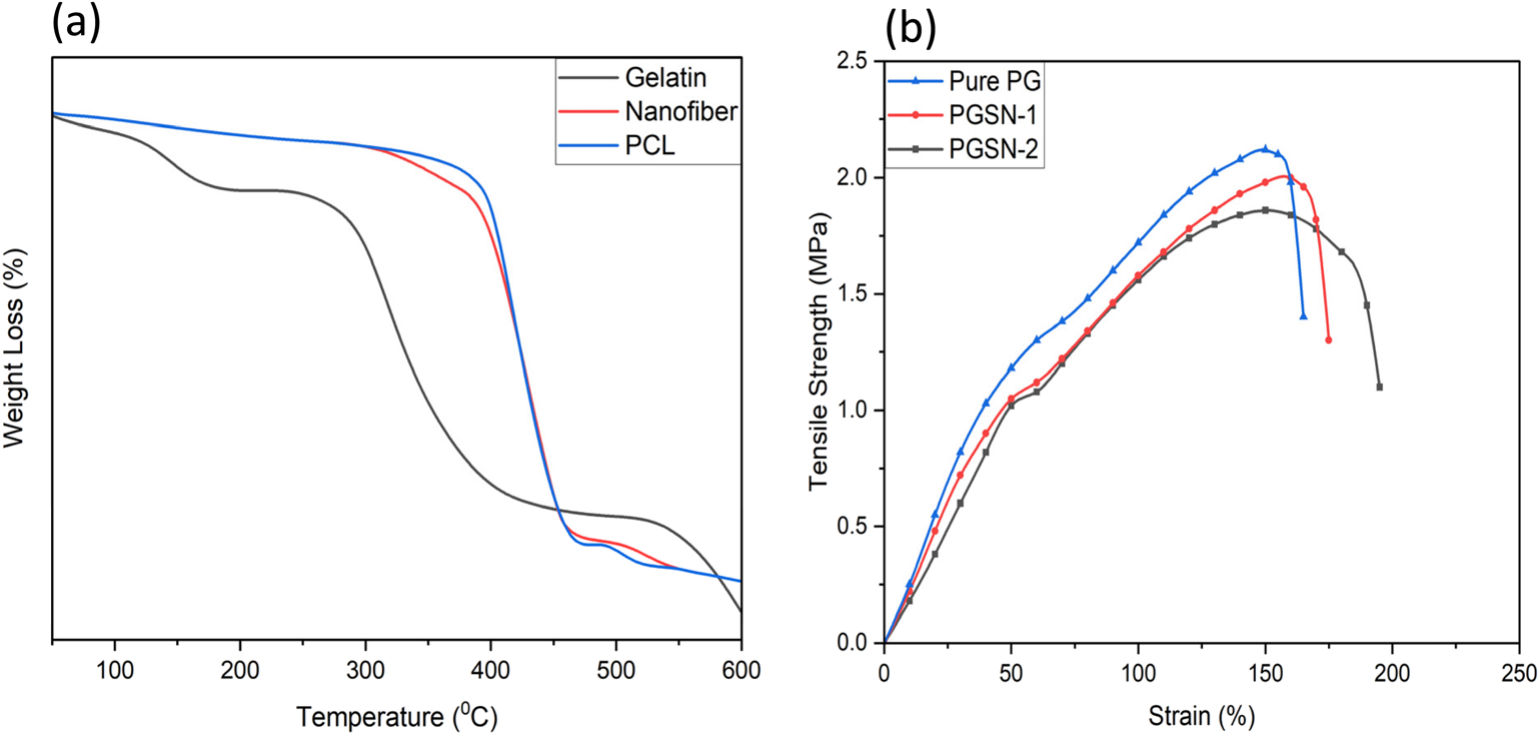
(a) TGA curves of gelatin, PCL, and nanofibrous mat. (b) Stress–strain behavior of pure PG, PGSN-1, and PGSN-2 samples.

TGA data provided detailed insights into the thermal degradation characteristics of different samples, as shown in Fig. 5a. PCL underwent a single-stage thermal degradation between 372 °C and 457 °C, suggesting only one significant degradation event in this temperature span. On the contrary, GEL degraded in two stages; the initial degradation stage was between 46 °C and 218 °C, and the second stage was from 247 °C to 590 °C. These phases suggested disintegration of the GEL constituents, whereby the first stage involved the loss of moisture content and low-molecular-weight substances, and the second phase involved dissolution of the polymer backbone.

In the case of the developed nanofibrous mat, the degradation of the material by thermal means was one stage and in a temperature span of 390 °C to 540 °C. This indicated that the addition of the extract to the PCL/GEL nanofiber matrix affected the overall thermal degradation character, resulting in a smoother degradation profile. This transition to a single-stage degradation suggested that the interactions between the composition of PCL, GEL, and the extract yielded a change in the thermal stability and degradation behavior of the material, which improved the structural integrity of the nanofibers in general.

Therefore, TGA showed that the structure and composition of nanofibers could influence the thermal degradation characteristics of the synthesized nanofibrous mat significantly, which suggested effective incorporation of the extract into the polymer network. This change in thermal stability rendered the nanofibrous mat especially applicable in biomedical applications because it exhibited higher material stability at physiological conditions. Hence, it would be beneficial in applications such as drug delivery, wound healing, and tissue engineering.^80^

### 3.4 Moisture management test

**Fig. 6:**
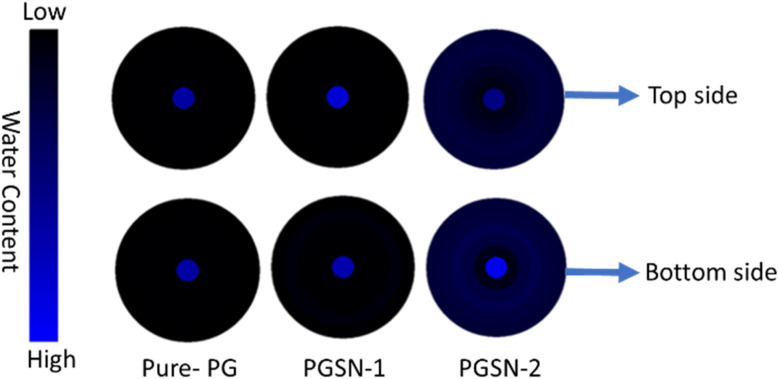
Water distribution on the top and bottom surfaces of Pure-PG, PGSN-1, and PGSN-2 samples (schematic).

All measurements were performed in triplicate (*n* = 3) and expressed as mean ± standard deviation (Table 2 and Fig. 6). The pure-PG sample had a low ability to absorb moisture, a low spreading rate, a negative AOTI, and a very low OMMC, indicating that it was water-repellent and did not transport moisture. These properties are also undesirable if used in biomedical applications because inappropriate moisture management will result in fluid retention and an inappropriate microenvironment at the tissue–material interface. On the contrary, samples of PGSN-1 and PGSN-2 showed much better results in terms of moisture management, as indicated by increased absorption rates, increased spreading speed, and markedly positive AOTI values, which suggested the unidirectional movement of moisture from the top to the bottom surface. Among the samples, PGSN-2 exhibited the fastest wetting, the greatest absorption capacity and the greatest OMMC value, thereby highlighting superior capability to absorb, distribute, and transport excess fluid without backflow. The improved moisture management properties observed with SN incorporation could be attributed to the presence of hydrophilic phytoconstituents in the extract. These polar functional groups can enhance the affinity of the nanofiber surface towards water molecules, leading to increased moisture absorption and transport. Furthermore, the incorporation of SN extract may have influenced fiber morphology and surface characteristics, which can also contribute to improved moisture handling.

**Table 2:** Moisture management properties (mean ± SD)

Sample	Wetting top (s)	Wetting bottom (s)	Absorption top (g s^−1^)	Absorption bottom (g s^−1^)	Spreading top (mm s^−1^)	Spreading bottom (mm s^−1^)	AOTI	OMMC	AATCC classification
Pure-PG	5.14 ± 0.21	7.34 ± 0.28	5.60 ± 0.30	7.74 ± 0.35	0.46 ± 0.02	0.49 ± 0.03	−3.84 ± 0.25	0.05 ± 0.01	Water repellent
PGSN-1	16.60 ± 0.45	6.63 ± 0.30	7.80 ± 0.40	40.26 ± 1.20	0.24 ± 0.01	0.61 ± 0.04	513.1 ± 5.5	0.41 ± 0.02	Water penetration
PGSN-2	3.52 ± 0.18	2.60 ± 0.15	22.20 ± 0.90	49.06 ± 1.50	1.06 ± 0.05	1.16 ± 0.06	562.3 ± 6.2	0.68 ± 0.03	Water penetration

An “ideal” wound dressing should maintain a moist environment to facilitate cell migration, proliferation, and tissue regeneration, while also allowing excess exudate to be absorbed and transported away from the wound site. Appropriate moisture balance is essential to prevent dehydration as well as the maceration of surrounding tissues. Our developed nanofibrous mats exhibited favorable moisture management properties. Incorporation of the SN extract improved the absorbency and liquid transport behavior, indicating the ability to maintain an optimal moist environment. These properties suggest that the fabricated nanofibers are suitable for wound healing applications. Such properties are especially beneficial for biomedical uses (*e.g.*, wound dressing, tissue-engineering scaffolds, and biomedical textiles in contact with the skin), where exudate control, maceration prevention and creation of an optimal moist environment are essential for tissue regeneration and healing.^81^ Hence, the increased moisture regulation capacity of PGSN samples (in particular PGSN-2) indicated that they would be prospective candidates in next-generation biomedical applications that necessitate efficient fluid handling and sustained physiological comfort.^82^ One-way ANOVA indicated significant differences (*p* < 0.05) in key moisture management parameters, suggesting that the SN extract enhanced the moisture transport of nanofibrous mats.

### 3.5 Antibacterial assay

**Fig. 7:**
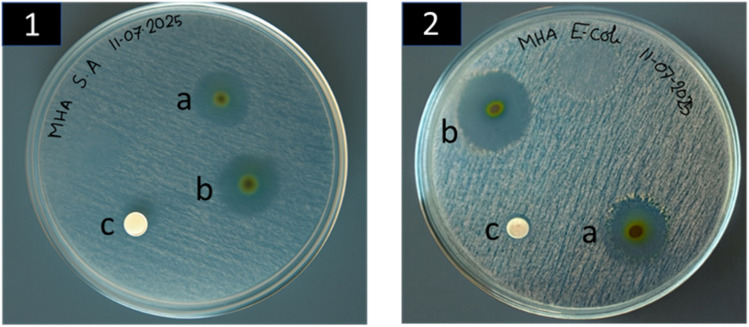
Formation of inhibition zones against (1) Gram-positive bacteria and (2) Gram-negative bacteria for (a) PGSN-1, (b) PGSN-2, and (c) Pure-PG samples.

The Kirby–Bauer disk diffusion method was used to evaluate the antibacterial efficacy of three nanofibrous mat samples (Pure PG, PGSN-1, and PGSN-2) against Gram-positive (*Staphylococcus aureus*) and Gram-negative bacteria (*Escherichia coli*), as shown in Fig. 7. The Pure-PG sample exhibited no inhibitory zones against either bacterial type, signifying that the absence of the extract in the nanofibers failed to elicit antibacterial activity. PGSN-2 exhibited higher antibacterial activity with inhibition zones of 25.7 ± 0.6 mm (Gram-positive) and 30.3 ± 0.6 mm (Gram-negative) compared with PGSN-1 (23.3 ± 0.6 mm and 28.3 ± 0.6 mm, respectively), with one-way ANOVA showing significant differences for Gram-positive and Gram-negative bacteria (*p* < 0.05). These results indicated that the addition of the SN extract to PCL/GEL nanofibers substantially increased their antibacterial properties, with the PGSN-2 sample showing the strongest effect.

This increase in antibacterial activity with extract concentrations could have been because an increase in bioactive chemicals in the extract likely increased the ability of the material to inhibit bacterial growth.^83^ Results revealed that incorporation of the SN extract in PCL/GEL nanofibers boosted their antibacterial property at the higher concentrations of the extract, which qualifies them to be used in biomedical applications if antibacterial effects are needed, such as wound healing or drug delivery.^84^

### 3.6 Cytotoxicity assay

**Fig. 8:**
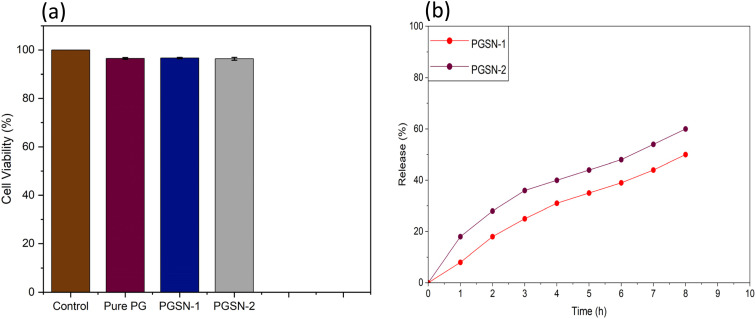
(a) Cell viability (%) of control, Pure PG, PGSN-1, and PGSN-2 samples. (b) Cumulative release profiles of the SN extract from PGSN-1 and PGSN-2 nanofibers.

*In vitro* cytotoxicity tests use cultured cells to assess the safety of biomaterials and drugs. Such tests measure cell metabolism. It has been demonstrated that the composition of scaffolds and the type of assay used can affect the results.^85^ Fabricated nanofiber mats were cultured in Vero cells (African green monkey kidney epithelial cells). They were exposed to nanofiber mats for over 48 h to determine the presence of cytotoxic effects, as shown in Fig. 8a. The viability of the Vero cells on the fabricated nanofiber mats was evaluated based on cytotoxicity data.

The control sample (which was not exposed to any extract) had 100% cell viability, so these cells were healthy and not affected by the test conditions. Cytotoxicity results demonstrated that Pure PG, PGSN-1 and PGSN-2 nanofibers exhibited high cell viability with minimal variation among replicates. Pure PG and PGSN-1 showed cell viability of 96.52 ± 0.38% and 96.67 ± 0.26%, respectively, whereas PGSN-2 exhibited 96.37 ± 0.58%, indicating consistent performance across three independent measurements (*n* = 3). This slight reduction in cell viability compared with that of the control could be attributed to the presence of bioactive compounds in the SN extract. However, cell viability >90% is generally considered safe for biomedical applications, confirming the non-toxic nature of our developed nanofibers. There was no significant difference between the two groups (*p* > 0.05), indicating comparable cytocompatibility.^86^

Hence, the developed nanofibers, including the extract, were non-toxic and would be safe in biomedical practice, including drug delivery and wound healing. Therefore, the engineered nanofiber mats could find application in biomedical practice without adversely affecting cells or tissues.^87^

### 3.7 Tensile strength

The tensile test was essential to evaluate the mechanical strength and strain behavior of the electrospun nanofibrous mats for biomedical applications. These parameters ensure that the material can withstand mechanical stress while maintaining sufficient flexibility for practical use.

The tensile behavior of the electrospun nanofibrous mats (Pure PG, PGSN-1, and PGSN-2) is presented in Fig. 5b. Pure PG exhibited the highest tensile strength (2.10 ± 0.05 MPa) at approximately 150–155% strain, demonstrating superior load-bearing capacity due to the dominant contribution of the PCL matrix. In contrast, PGSN-1 and PGSN-2 showed a slightly reduced tensile strength of 2.00 ± 0.06 MPa and 1.85 ± 0.07 MPa, respectively, which could be attributed to the incorporation of the SN extract that may weaken intermolecular interactions within the nanofibrous structure. Pure PG exhibited an elongation at break of 160 ± 5%, whereas PGSN-1 and PGSN-2 showed an increased strain of 170 ± 6% and 200 ± 8%, respectively. This indicated that the addition of the SN extract enhanced the extensibility of the nanofibrous mats, likely due to a plasticizing effect that facilitates polymer chain mobility. Stress–strain curves further revealed that all samples underwent deformation followed by failure, with PGSN-2 showing the highest strain at break, indicating improved ductility. These results demonstrated a trade-off between tensile strength and strain, whereby incorporation of the SN extract reduced the strength but significantly improved flexibility. Such a balance is important for biomedical applications, where mechanical stability and adaptability are required.

All measurements were performed in triplicate (*n* = 3), and the results are expressed as mean ± standard deviation (SD). Statistical significance among samples was analyzed using one-way ANOVA, with *p* < 0.05 considered significant.

### 3.8 Release properties of the extract

The *in vitro* release profiles of the SN extract from the electrospun nanofibrous mats (PGSN-1 and PGSN-2) are presented in Fig. 8b. Release behavior demonstrated a time-dependent increase in cumulative release for both samples, indicating effective diffusion of bioactive compounds from the nanofibrous matrix. Initially, both samples exhibited a relatively faster release within the first few hours, which could be attributed to the release of extract molecules located near or on the fiber surface. Specifically, PGSN-2 showed a higher initial release (18% at 1 h) compared with PGSN-1 (8%), suggesting a greater availability of surface-associated extract or a more open fiber structure. As time progressed, the release rate gradually decreased, transitioning into a sustained-release phase. This behavior indicated that the remaining extract was primarily entrapped within the polymer matrix and diffused slowly over time. At 8 h, PGSN-2 reached a cumulative release of ∼60%, whereas PGSN-1 exhibited a comparatively lower release of ∼50%. The higher release observed by PGSN-2 suggested enhanced diffusion, which could be attributed to differences in extract loading, fiber morphology, or intermolecular interactions within the nanofibrous network. In contrast, the relatively slower release of PGSN-1 indicated stronger interaction between the extract and polymer matrix, resulting in more controlled release.

Both samples exhibited a combination of initial burst release followed by sustained release, which is highly desirable for wound dressing applications. The initial release helps to provide immediate antibacterial action, while the sustained release ensures prolonged therapeutic effect. Among the samples, PGSN-2 showed a faster release profile, whereas PGSN-1 demonstrated comparatively better controlled-release characteristics.

## 4 Conclusions

Antibacterial electrospun nanofibrous mats based on PCL, GEL, and an SN extract were fabricated and evaluated for biomedical applications. Addition of the SN extract also had a considerable effect on the structural, functional and biological characteristics of the nanofibrous mats. SEM revealed the creation of homogenous and bead-less nanofibers, with a decreasing average fiber diameter as the extract concentration increased, which suggested an enhancement in fiber morphology. The developed nanofibrous mats also exhibited adequate tensile strength and elongation at break, ensuring good mechanical stability and flexibility. Furthermore, *in vitro* release analysis demonstrated a controlled and sustained release behavior of SN extract. The MMT showed that extract-loaded mats had improved wetting behavior, increased rate of absorption, positive one-way moisture transport, and improved moisture management capacity compared to those of the control sample. Antibacterial tests indicated that mats with the SN extract contained high levels of inhibitory activity against Gram-positive and Gram-negative bacteria, with a greater antibacterial effect found with increased extract concentrations. Moreover, *in vitro* cytotoxicity tests revealed excellent biocompatibility because cell viability was >95%, which denoted that the manufactured mats were safe to be applied in biomedical practice.

The resulting PCL/GEL/SN electrospun nanofibrous mats could be used as advanced wound dressing materials because of the combined antibacterial effect, effective moisture regulation and biocompatibility. However, our research was confined to *in vitro* analyses, and *in vivo* wound healing was not examined. Further studies are required to investigate detailed *in vivo* wound healing performance, long-term biodegradation, and optimized controlled release kinetics to validate the clinical applicability of these nanofibrous mats.

## Author contributions

Amanullah conceived and designed the study, carried out the experimental work, and performed data analyses/interpretation. He prepared the original draft of the manuscript and coordinated the overall research activities. Mainul Islam contributed to the conception and design of the study and assisted in data analyses/interpretation. He critically revised the manuscript for important intellectual content. Mohammed Mustafizur Rahman contributed to the development of methodology, interpretation of results, and preparation of figures and graphical elements. He also critically reviewed and revised the manuscript. Mohammed Farhad Hossain contributed to the study design, provided supervision during the research work, and supported data interpretation. He critically revised the manuscript and provided intellectual guidance throughout the study. All authors reviewed and approved the final version of the manuscript and agree to be accountable for all aspects of the work, ensuring that any questions related to the accuracy or integrity of any part of the work are appropriately investigated and resolved.

## Conflicts of interest

The authors declare no conflicts of interest related to the research, authorship, or publication of this article.

## Data Availability

All data generated or analyzed during this study are included in the published article.
